# Atypical primary biliary cholangitis results in vanishing bile duct syndrome with cutaneous xanthomas: a case report

**DOI:** 10.1186/s13000-022-01228-1

**Published:** 2022-07-04

**Authors:** Yuebo Jia, Lin Liu, Baocheng Deng, Yu Huang, Jiaqi Zhao, Guang Bai

**Affiliations:** 1 Graduate Studies, Liaoning University of Traditional Chinese Medicine, Shenyang, China; 2 Department of Gastroenterology, Affiliated Hospital of Liaoning University of Traditional Chinese Medicine, Shenyang, China; 3 The First Affiliated Hospital, China Medical University, Shenyang, China

**Keywords:** Vanishing bile duct syndrome, Primary biliary cholangitis, Cutaneous xanthomas, Case report

## Abstract

**Background:**

Vanishing bile duct syndrome (VBDS) is a rare but potentially severe acquired chronic cholestatic liver disease. Bile duct deficiency is a reduction of bile ducts in the liver, which can eventually lead to cholestatic liver disease and progress to biliary cirrhosis. Primary biliary cholangitis (PBC) is one of the causes of bile duct deficiency. In addition, 75% of PBC patients may have dyslipidemia, and in case of secondary dyslipidemia, cutaneous xanthomas may occur.

**Case summary:**

A 49-year-old woman was admitted with jaundice and multiple subcutaneous nodules. She received diagnosis of autoimmune liver disease 2 years before. Although she was treated with liver-protecting drugs, such as Essentiale and ursodeoxycholic acid, jaundice occurred repeatedly, and the color of her skin was becoming darker and more yellow.

**Conclusion:**

This case highlights that the positivity of ANA that in PBC have a well diagnostic and prognostic significance and antinuclear antibodies giving the ‘multiple nuclear dots’ or the ‘rim-like/membranous’ pattern scan ca diagnose primary biliary cirrhosis accurately. Since the liver biopsy of PBC alone may not be sufficient to establish the diagnosis, serum antibodies should also be examined. PBC can also lead to intrahepatic cholestasis, which can cause dyslipidemia and cutaneous xanthomas.

## Introduction

Vanishing bile duct syndrome (VBDS) is defined as a finding in specimens containing portal areas; absence of interlobular bile duct is found in more than 50% of the portal area. The etiology of VBDS is variable, including drugs, immunity, congenital malformation, tumor, infection, and ischemia and hypoxia. Among them, immune factors causing biliary system injury are an important mechanism of VBDS [1]. Diagnosis is improved by immunostaining for CK7 and CK19 in liver biopsy specimens, both of which identify bile duct components [2]. VBDS is not an independent disease, but rather a pathologic feature of continuous progressive destruction of intrahepatic bile ducts due to various factors. Some patients with the absence of intrahepatic bile ducts may have symptoms of cholestasis, such as jaundice, itching, and fatigue, while others may be asymptomatic [3]. Here, we described a case of VBDS diagnosed by liver biopsy and serological analysis, finally diagnosed with PBC, who also had cutaneous xanthomas [4].

## Case presentation

### Chief complaints

A 49-year-old woman presented to the TCM Gastroenterology Clinic of our hospital complaining of jaundice and multiple subcutaneous nodules that had been lasting for 2 years.

### History of present illness

The patient’s symptoms started 2 years ago with manifestations of jaundice and multiple subcutaneous nodules, and worsened over the last 3 days. Two years ago, she received diagnosis of autoimmune hepatitis (AIH), but the specific type was unknown. Although she received Polyene phosphatidylcholine 228 mg oral tid and ursodeoxycholic acid 250 mg oral qid, jaundice occurred repeatedly. The patient received a detailed medical examination and treatment at the current hospital.

### History of past illness

The patient had type 2 diabetes for 4 years, and had been using metformin sustained-release tablets intermittently to control blood glucose.

### Personal and family history

The patient did not abuse alcohol or substances. There was no family history of liver disease.

### Physical examination

**Fig. 1:**
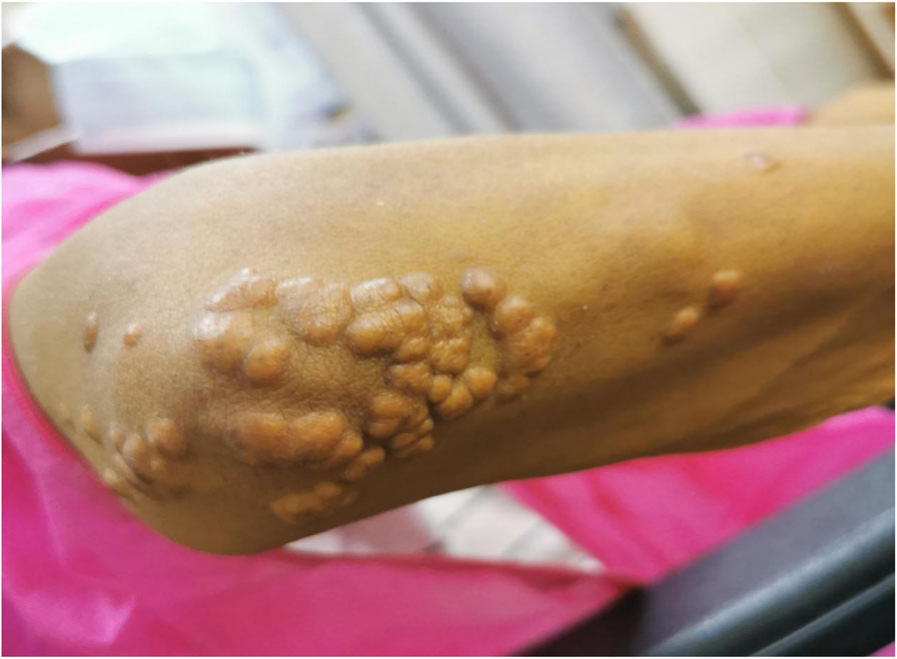
Numerous xanthomas in the patient. Histopathological assessment of a subcutaneous nodule from the elbow joint revealed that it was xanthoma

The patient had yellow skin and icteric sclera, and her elbow, wrist, finger joints, and metacarpophalangeal joints presented multiple, scattered, soft non-tender subcutaneous nodules (Fig. 1). The inferior border of the spleen exceeded the lower costal margin by 3 cm; there was sternal tenderness and L2–L3 spinal percussive pain.

### Laboratory examinations

Blood samples revealed alanine aminotransferase (ALT) level of 103 U/L(normal range,5 to 35), serum aspartate aminotransferase (AST) level of 199 U/L(normal range,8 to 40), γ-glutamyl-transpeptidase (γ-GGT) level of 824 U/L(normal range,7 to 32), alkaline phosphatase (ALP) level of 1879 U/L(normal range,70 to 150), prothrombin time (PT) of 21.2 s(normal range,10 to 14), international normalized ratio (INR) of 1.85(normal range,0.8 to 1.24), activated partial thromboplastin time (APTT) of 52.8 s(normal range,23 to 35), total bilirubin (TBIL) level of 515.76 μmol/L(normal range,3.42 to 20.52), direct bilirubin (DBIL) level of 249.88 μmol/L(normal range,<=6.91), indirect bilirubin (IBIL) level of 265.88 μmol/L(normal range,2 to 15.22), total bile acid (TBA) level of 255.5 μmol/L0.(normal range,14 to 9.66), and free triiodothyronine (FT3) level of 1.83 pmol/L(normal range,3.1 to 6.8). Blood tests showed no obvious abnormalities. Triglyceride (TG) level of 5.28 mmol/L(normal range,0.7 to 1.7), total cholesterol (CHOL) level of 18.17 mmol/L(normal range,<=5.2),HDL cholesterol(HDL-C) level of 1.56 mmol/L(normal range > =1.03), LDL-Cholesterol (LDL-C) level of 0.55 mmol/L(normal range < − 3.62), C-reactive protein (CRP) level of 103.59 mg/L(normal range,<=8), IgA level of 4.16 g/L(normal range,2.01 to 2.69), and IgG level of 15.31 g/L(normal range,11.52 to 14.22) were found. Antibody screening revealed positive antinuclear antibody (ANA), antimitochondrial (AMA), anti-mitochondrial M2 (M2-3E), and hepatitis E IgG (HEV-IgG) antibodies. Tumor markers showed positive CA12–5 (37.28 U/mL)(normal range,<=35) and CA72–4 (24.45 U/mL)(normal range,<=6.9).

### Imaging examinations

Computed tomography (CT) scan of the whole abdomen revealed hepatosplenomegaly, widened portal veins, hepatic cysts, cholecystolithiasis, and ascites. 3-D CT scan showed multiple old fractures of right ribs. Her lumbar spine magnetic resonance (MR) images showed L1–L5 vertebral compression fractures.

### Pathological examination

**Fig. 2:**
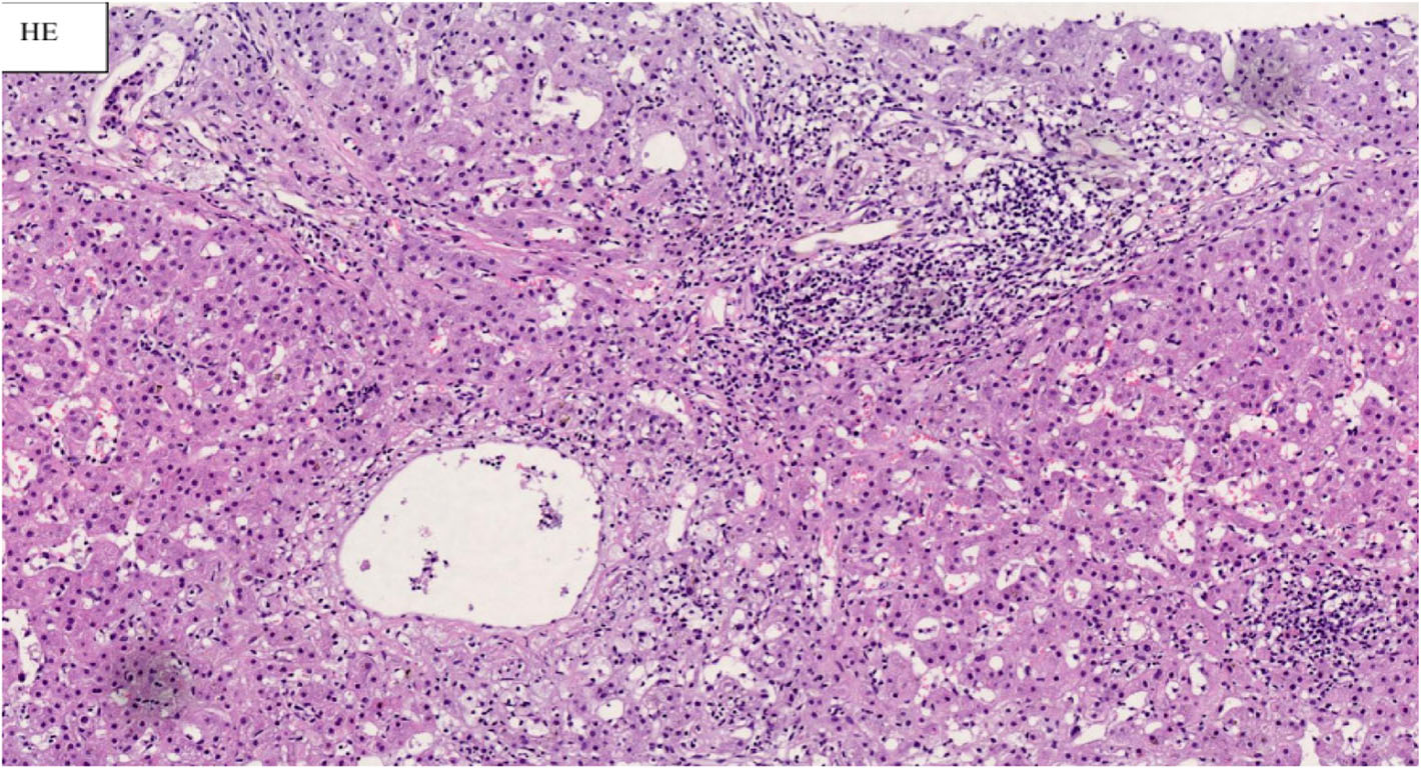
Hepatic tissue of Hematoxylin and eosin staining. Hematoxylin and eosin staining showed 25 fibrosis expanded small and medium portal areas and an incomplete large one

**Fig. 3:**
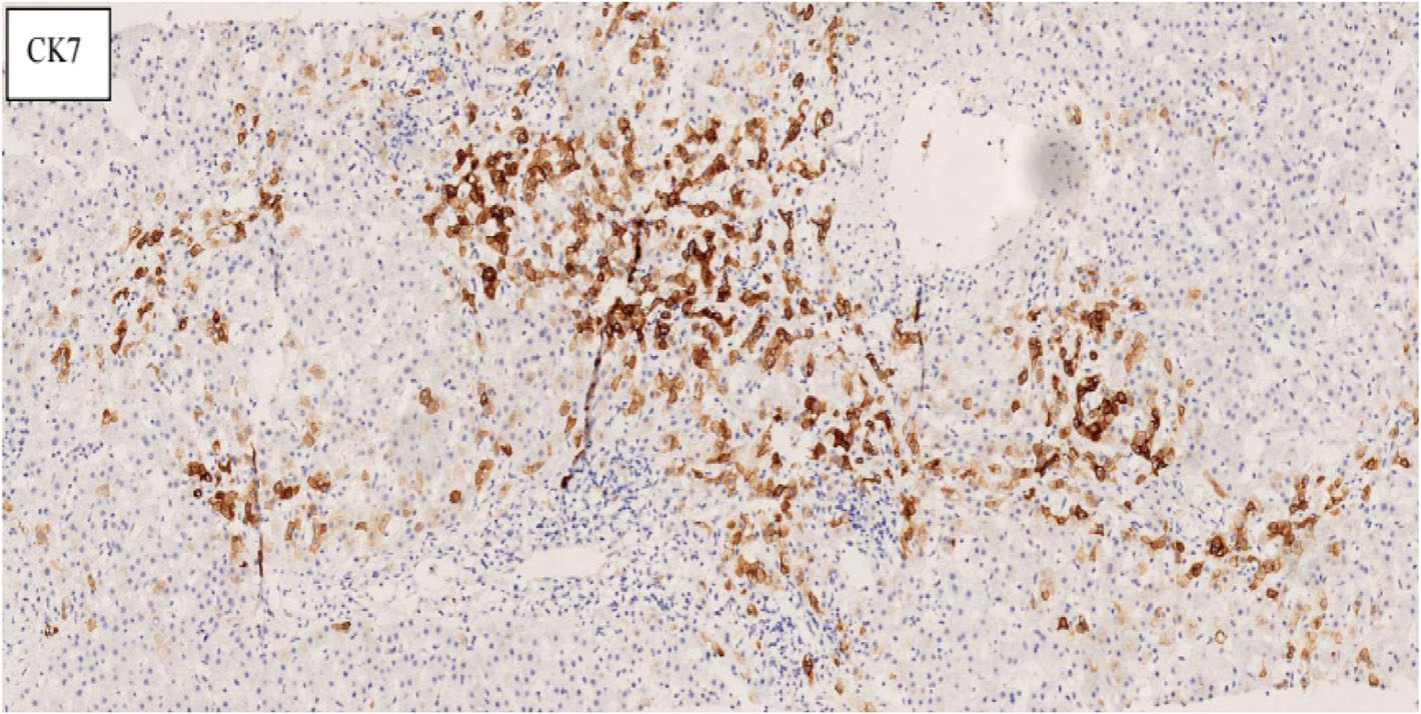
Hepatic tissue of IHC, immunohistochemistry for CK7. It can improve the diagnosis of VBDS and PBC more reliable

Percutaneous liver needle biopsy revealed 25 fibrosis expanded small and medium portal areas and an incomplete large one (Figs. 2, 3). Among them, the epithelium of small bile ducts was not complete in three large and incomplete large portal areas, and lymphocytes infiltration was observed. Hepatocyte swelling and bile salt accumulation were noted, but no arterioles accompanying small bile ducts in other portal areas were found. Multiple confluent fields of necrosis in lobules, collapse of reticular scaffolds, and perisinusoidal fibrosis were also found. Some hepatocytes had cholestasis, and some hepatic sinusoids nonobstructively expanded with aberrant erythrocyte morphology.

Histopathological assessment of a subcutaneous nodule from the elbow joint revealed that it was xanthoma.

### Diagnostic procedure

Based on the patient’s pathological examination, the diagnosis of VBDS was made. Histological lesions were graded G2S2 as per the modified Scheuer score. Combined with the laboratory examinations, she received the diagnosis of PBC. After symptomatic treatment, we discharged the patient on the 25th day of hospitalization with the following serum results: AST 128 U/L, ALT 51 U/L, γ-GGT 670 U/L, TBIL 515.76 μmol/L, DBIL 249.88 μmol/L, IBIL 268.88 μmol/L, and ALP 720 U/L.

The patient continued to take ursodeoxycholic acid 250 mg oral qid and fenofibrate 200 mg oral qd after discharge.

## Final diagnosis

Liver biopsy pathology only: Vanishing bile duct syndrome.G2S2.This suggested liver fibrosis but not cirrhosis.

Clinical diagnosis combined with liver pathology and serum antibodies: primary biliary cholangitis (PBC), phaseII (Inflammation and/or fibrosis limited to and around the portal area);

## Treatment

Treatment included ursodeoxycholic acid 250 mg oral qid and fenofibrate 200 mg oral qd,

in combination with calcium 600 mg and vitamin D 500 units oral tid.

## Outcome and follow-up

Based on the results of 25-day-long treatment, the patient responded poorly to ursodeoxycholic acid. Considered low drug response rate, jaundice did not abate. Liver transplantation is considered the only effective treatment for end-stage PBC.However in low income patients, liver transplantations will not be performed.

## Discussion

The etiology of VBDS is diverse, including congenital and genetic diseases, ischemia and hypoxia, tumors, infections, immune disorders, idiopathic adult intrahepatic bile duct deficiency, and other factors. Biliary system injury caused by immunological factors is an important mechanism of VBDS, and PBC is an autoimmune liver disease. With the progression of PBC, small bile ducts affected by immunological process may gradually disappear, thereby leading to VBDS. Meanwhile, given that cholestasis affects lipid metabolism, individuals with PBC may show elevated cholesterol levels and develop cutaneous xanthomas.

In this case, liver biopsy indicated disappearance of intrahepatic bile ducts but did not find well-defined pathological features of PBC. Yet, PBC is one of the causes of disappearance of intrahepatic bile ducts, and it was confirmed via serological tests showing positive AMA and AMA2-3E antibodies. Therefore, for more accurate diagnosis, combining multiple assessment methods is needed in clinical practice.

## Conclusion

This case highlights that the positivity of ANA that in PBC have a well diagnostic and prognostic significance [5] and antinuclear antibodies giving the ‘multiple nuclear dots’ or the ‘rim-like/membranous’ pattern scan ca diagnose primary biliary cirrhosis accurately [6]. Since the liver biopsy of PBC alone may not be sufficient to establish the diagnosis, serum antibodies should also be examined. PBC can also lead to intrahepatic cholestasis, which can cause dyslipidemia and cutaneous xanthomas.

Ursodeoxycholic acid should be used first for patients with VBDS caused by PBC [7, 8]; for the patients with high lipids or even cutaneous xanthomas, fenofibrate should be added. Many patients with VBDS eventually require liver transplantation due to poor capillary regeneration.

## Data Availability

All data, models, and code generated or used during the study appear in the submitted article.
